# Retention in care outcomes for HIV pre-exposure prophylaxis implementation programmes among men who have sex with men in three US cities

**DOI:** 10.7448/IAS.19.1.20903

**Published:** 2016-06-13

**Authors:** Philip A Chan, Leandro Mena, Rupa Patel, Catherine E Oldenburg, Laura Beauchamps, Amaya G Perez-Brumer, Sharon Parker, Kenneth H Mayer, Matthew J Mimiaga, Amy Nunn

**Affiliations:** 1 Division of Infectious Diseases, The Miriam Hospital, Brown University, Providence, RI, USA; 2 Division of Infectious Diseases, University of Mississippi Medical Center, Jackson, MS, USA; 3 Division of Infectious Diseases, Washington University in St. Louis, St. Louis, MO, USA; 4 Department of Epidemiology, Harvard TH Chan School of Public Health, Boston, MA, USA; 5 Department of Sociomedical Sciences, Columbia University, New York, NY, USA; 6 Department of Social Work, North Carolina Agricultural and Technical State University, Greensboro, NC, USA; 7 The Fenway Institute, Fenway Health, Boston, MA, USA; 8 Department of Medicine, Beth Israel Deaconess Medical Center, Boston, MA, USA; 9 Department of Global Health and Population, Harvard School of Public Health, Boston, MA, USA; 10 Department of Epidemiology, Brown University School of Public Health, Providence, RI, USA; 11 Department of Behavioral and Social Sciences, Brown University School of Public Health, Providence, RI, USA

**Keywords:** pre-exposure prophylaxis, implementation, men who have sex with men, HIV

## Abstract

**Introduction:**

Despite the efficacy of pre-exposure prophylaxis (PrEP) in preventing HIV transmission, few studies have evaluated PrEP use and retention in care outcomes in real-world settings outside of clinical trials.

**Methods:**

Data were collected from PrEP clinical care programmes in three mid-size US cities: Providence, Rhode Island (RI); Jackson, Mississippi (MS); and St. Louis, Missouri (MO). We assessed the demographic and social characteristics of patients prescribed PrEP and documented their insurance and copayment experiences. We assessed retention in PrEP care at three and six months. Multivariate analyses were used to predict retention in care among men who have sex with men (MSM). HIV acquisition among the cohort was also assessed.

**Results:**

A total of 267 (RI: 117; MS: 88; MO: 62) patients were prescribed PrEP; 81% filled prescriptions (RI: 73%; MS: 82%; MO: 94%; *p*<0.001). Patients in MS and MO were more commonly African American than in RI (72% and 26% vs. 7%, respectively), but less frequently Latino (2% and 3% vs. 24%, respectively). More patients reported living below the federal poverty line in MS (52%) compared to MO (23%) and RI (26%). Most patients were MSM (RI: 92%; MS: 88%; MO: 84%). The majority of MSM reported recent condomless anal sex (RI: 70%; MS: 65%; MO: 75%). Among 171 patients prescribed PrEP at least six months beforehand, 72% were retained in care at three months (RI: 68%; MS: 70%; MO: 87%; *p*=0.12) and 57% were retained in PrEP care at six months (RI: 53%: MS: 61%; MO: 63%; *p*=0.51). Insurance status and medication costs were not found to be significant barriers for obtaining PrEP. Three patients became infected with HIV during the six-month period after being prescribed PrEP (1.1%; 3/267), including one in RI (suspected acute HIV infection), one in MO (confirmed poor adherence) and one in MS (seroconverted just prior to initiation).

**Conclusions:**

PrEP initiation and retention in care differed across these distinct settings. In contrast, retention in PrEP care was consistently suboptimal across sites. Further research is needed to identify the individual, social and structural factors that may impede or enhance retention in PrEP care

## Introduction

HIV pre-exposure prophylaxis (PrEP) is a biomedical HIV prevention modality that entails the daily use of the single-tablet antiretroviral medication emtricitabine (FTC) and tenofovir disoproxil fumarate (TDF) by uninfected individuals at risk for HIV infection. PrEP's efficacy in preventing HIV acquisition has been demonstrated in randomized controlled trials [1–5] and open-label studies [6]. These studies also demonstrated that better adherence dramatically enhances PrEP's efficacy [7,8].

Data is beginning to emerge about PrEP implementation in real-world settings [9–14]. Although initial reports of PrEP implementation indicated slow uptake [9,10], PrEP coverage is expanding across the United States [15,16]. A recent study found that PrEP uptake in a primary care setting reached individuals at high risk for HIV acquisition, and no new infections were reported, underscoring both the feasibility of PrEP delivery and its effectiveness in reducing HIV acquisition in real-world clinical settings [11,17]. Although over 13,000 people have accessed PrEP in the United States, a figure that continues to increase [18], much of the peer-reviewed literature about PrEP implementation programmes is based on Centers for Disease Control and Prevention (CDC) demonstration projects that provide PrEP free of charge in major metropolitan areas [11–13]. Although evidence is beginning to emerge in some health systems structures in the United States, such as the Kaiser Permanente cohort in Northern California [11], little is known about PrEP implementation in other clinical contexts.

TDF/FTC, the only FDA-approved PrEP medication for HIV primary prevention, costs approximately $1400/month. Cost has been cited as an important barrier that may limit PrEP use [10,19–21]. Previous efficacy trials and open-label PrEP programmes provided medication free of charge [12,22], which may facilitate PrEP use [9]. In order to maximize access to PrEP, economic considerations related to PrEP uptake, including how patients pay for PrEP, need to be addressed, and barriers and facilitators of ongoing PrEP care need to be understood. Additional information is also needed to understand how insurance coverage and industry-sponsored assistance programmes impact PrEP uptake and retention in care.

To add to the growing literature on PrEP implementation, we present results from three PrEP implementation programmes in Providence, RI, Jackson, MS, and St. Louis, MO, where PrEP was provided in clinical settings. We present demographic and behavioural data for individuals prescribed PrEP, as well as adherence and retention in care outcomes associated with this three-site PrEP implementation programme.

## Methods

### Clinical sites and eligibility

In 2014, PrEP programmes were established at three clinics in Providence, RI, Jackson, MS and St. Louis, MO. These three clinics were the first established PrEP programmes in each state. In Rhode Island, PrEP was offered at a sexually transmitted disease (STD) and HIV prevention clinic. Other patients were referred from the on-site HIV clinic and other providers. In Mississippi, PrEP was offered at a lesbian/gay/bisexual/transgender (LGBT) outpatient clinic. PrEP patients in Mississippi were referred from ongoing research studies, the state STD clinic and other providers. In Missouri, PrEP was offered at an infectious diseases specialty clinic where the majority of referrals were from outpatient medical providers.

Individuals presenting for care were evaluated for behaviours associated with HIV acquisition and, if indicated, educated and prescribed PrEP in accordance with CDC guidelines [23]. PrEP was offered to men who have sex with men (MSM) reporting condomless anal intercourse, individuals in serodiscordant partnerships and other at-risk populations such as people who inject drugs. Demographic and behavioural data were reviewed for all patients prescribed PrEP from January 2014 through September 2015.

Patients were followed longitudinally every three months in accordance with current CDC PrEP guidelines. Among individuals prescribed PrEP, we assessed rates of PrEP initiation (confirmed to have started the medication), retention in PrEP care at three and six months, and HIV seroconversion. Retention in care was defined as presenting for PrEP clinical services at three-month intervals. Patients were categorized as follows: 1) retained in care (still on PrEP); 2) discontinuing PrEP because it was no longer indicated for them; or 3) not retained in PrEP care (despite a continued indication for PrEP). Although every effort was made to evaluate patient outcomes every three months, a subset of patients did not keep scheduled visits but were known to continue taking PrEP. Patients who did not keep scheduled appointments but were in contact with clinic staff (e.g. by phone) and known to be on PrEP were considered retained.

### Data collection

We collected demographic, behavioural and insurance-related information during clinical visits. Demographic information included age, gender, race, ethnicity, referral source and insurance status. Behavioural information included the number and type of sexual partners, type of sexual behaviours, condom use and drug use. Type of insurance, requirement for prior authorizations and use of patient assistance programmes was reviewed for each patient. In the United States, types of insurance that cover PrEP care include private options, as well as public options (e.g. Medicaid, funded by the government).

Given that this assessment took place in the context of a real-world clinical programme, adherence was assessed by self-report. Patients were asked whether they had missed any doses in the previous seven and thirty days. Past-week adherence was reported based on taking four or more pills [24] or 100% adherence in the past seven days. Past-month adherence was reported based on having missed five or fewer pills or 100% adherence in the past month. The visit closest to the three- or six-month mark was considered the three- or six-month visit, respectively, as long as the visit was within one month of the appropriately timed visit cycle.

Baseline and follow-up characteristics for the study sample were described with means and standard deviations for continuous variables and proportions for categorical variables by study site and overall. A Fisher's exact test for categorical or Wilcoxon rank-sum test for continuous variables was used to test for differences in characteristics across sites. To assess factors associated with being prescribed PrEP and retention in care, a series of logistic regression models were built. The study sample for these models was restricted to individuals who had been prescribed PrEP at least six months prior and who were MSM, due to the overwhelming majority of MSM in the study population.

For each dependent variable (starting PrEP, being retained in care at three months and being retained in care at six months), a bivariate logistic regression model was first built for each independent variable (age, African American/black race *versus* any other race, university education or above *versus* below, income and no insurance *versus* any). Second, a multivariable model including all independent variables was run for each dependent variable. Models for retention in care at three and six months were restricted to individuals who had started PrEP. All analyses were conducted in Stata 13.1 (StataCorp, College Station, TX, USA).

This evaluation and study protocol were approved by the local institutional review boards.

## Results and discussion

### Demographics and behaviours

A total of 267 (Rhode Island: 117, Mississippi: 88, Missouri: 62) patients were prescribed daily PrEP between January 2014 and September 2015. In Rhode Island, most patients were referred from STD or HIV clinics, while most Mississippi patients were referred directly from the on-site LGBT clinic and most Missouri patients were referred from community organizations, HIV clinics or their primary care physician. Uptake was similar across sites, but demographics and insurance types differed (Table 1).

**Table 1:** Baseline characteristics of patients prescribed HIV pre-exposure prophylaxis in Providence, RI, Jackson, MS, and St. Louis, MO

	Providence, RI	Jackson, MS	St. Louis, MO	
	(*N*=117)	(*N*=88)	(*N*=62)	*p*
Age (mean, SD)	34.1 (11.4)	30.1 (10.3)	31.6 (8.4)	0.02
Gender				
Male	109 (93.2%)	79 (89.8%)	55 (88.7%)	0.65
Female	7 (6.0%)	8 (9.1%)	7 (11.3%)	
Transgender	1 (0.9%)	1 (1.1%)	0	
Race/ethnicity				
White/Caucasian	80 (68.4%)	21 (23.9%)	16 (25.8%)	<0.001
African American/black	8 (6.8%)	63 (71.6%)	38 (61.3%)	
Asian	3 (2.6%)	2 (2.3%)	2 (3.2%)	
Other^a^	26 (22.2%)	2 (2.3%)	6 (9.7%)	
Hispanic or Latino/a	28 (23.9%)	2 (2.3%)	2 (3.2%)	<0.001
Education				
Elementary school	3 (2.5%)	4 (4.7%)	2 (3.3%)	0.06
High school	44 (37.6%)	29 (33.7%)	18 (29.5%)	
College	50 (42.7%)	46 (53.5%)	23 (37.7%)	
Graduate school	20 (17.1%)	7 (8.1%)	18 (29.5%)	
Annual income, USD (mean, SD)	$47,179 ($53,423)	$19,122 ($21,862)	$37,522 ($35,345)	<0.001
Below $15,000/year (USD)	30 (25.6%)	47 (53.4%)	14 (22.6%)	<0.001
Insurance				
None	4 (3.4%)	44 (50.0%)	3 (4.8%)	<0.001
Private insurance	84 (71.8%)	38 (43.2%)	52 (83.9%)	
Medicare/Medicaid/other public	29 (24.8%)	6 (6.8%)	7 (11.3%)	
Prior authorization needed	8 (6.8%)	10 (11.4%)	10 (16.1%)	0.14
Participated in Gilead Patient Assistance Program	5 (4.3%)	57 (64.8%)	13 (21.0%)	<0.001
Copayment for medication a barrier	4 (3.4%)	26 (29.6%)	0	<0.001
Sexual risk behaviours^b^				
MSM	108 (92.3%)	77 (87.5%)	52 (83.9%)	0.22
MSF	5 (4.3%)	6 (6.8%)	3 (4.8%)	0.73
FSM	7 (6.0%)	4 (4.6%)	7 (11.3%)	0.27
Serodiscordant couple	37 (31.6%)	23 (26.1%)	22 (35.5%)	0.45
Condomless anal sex with another man^c^	76 (70.4%)	49 (65.3%)	39 (75.0%)	0.60
Anal sex with HIV-positive man^c^	27 (28.1%)	23 (30.7%)	17 (32.7%)	0.82
Substance use				
Alcohol use	91 (77.8%)	61 (69.3%)	56 (90.3%)	0.008
Injection drug use – ever	0	0	0	NA
Methamphetamine use – past three months	4 (3.4%)	1 (1.1%)	0	0.31
Popper (amyl nitrate) use – past three months	29 (24.8%)	5 (5.7%)	6 (9.7%)	<0.001
Referral source				
HIV clinic	23 (20.0%)	0	13 (21.0%)	<0.001
STD clinic	46 (39.3%)	22 (25.0%)	1 (1.6%)	<0.001
Primary clinic/LGBT clinic	0	27 (30.7%)	11 (17.7%)	<0.001
Other doctor	17 (14.5%)	3 (3.4%)	2 (3.2%)	0.006
Research study	3 (2.6%)	6 (6.8%)	0	0.07
PEP programme	7 (6.0%)	0	3 (4.8%)	0.05
Community organization	2 (1.7%)	0	17 (27.4%)	<0.001
Other	19 (16.2%)	30 (34.1%)	15 (24.2%)	0.01

a Includes multiracial, Cape Verdean, and Latino/Hispanic

b not mutually exclusive

c in the previous three months, restricted only to MSM.

RI: Rhode Island; MS: Mississippi; MO: Missouri; SD: standard deviation; USD: US dollars; MSM: men who have sex with men; MSF: men who have sex with females; FSM: females who have sex with males; STD: sexually transmitted disease; LGBT: lesbian/gay/bisexual/transgender; PEP: post-exposure prophylaxis.

Patients at all sites were overwhelmingly MSM. In Rhode Island, most patients (68%) were white and 24% were Latino, whereas 72% of patients in Mississippi and 61% in Missouri were African American. In Rhode Island and Missouri, only 4% and 5%, respectively, were African-American MSM under the age of 25, compared to 38% in Mississippi. A minority of patients were heterosexual women in serodiscordant relationships. Mean annual income varied widely between sites. Fifty-three percent of Mississippi patients lived below the federal poverty line, compared to 26% in Rhode Island and 23% in Missouri.

Reported risk behaviours were similar across sites. Among MSM patients, 70% in Rhode Island, 65% in Mississippi and 75% in Missouri reported recent condomless receptive anal sex. Approximately one-third of MSM at all three sites reported recent anal sex with men who were known to be HIV positive.

### Paying for PrEP

Whenever possible, insurance companies were billed for services and medications. Uninsured patients were enrolled in patient assistance programmes to cover clinical services and enrolled in the manufacturer's medication assistance programme to cover medication costs. A much higher fraction of Mississippi patients were uninsured compared to Rhode Island or Missouri. In Mississippi, 65% of patients participated in the medication assistance programme, compared to 21% in Missouri and 4% in Rhode Island (Table 1).

For insured patients, few challenges were encountered in billing for medications [14]. Few patients’ health plans required prior authorization for PrEP. Copayments for medications were barriers for 30% of Mississippi patients but only 3% of Rhode Island patients and none in Missouri. Copayments were generally covered by the manufacturer's copayment assistance programme and were not found to be a barrier to obtaining the medication for most patients. While billing for medical provider time and laboratory costs presented challenges for uninsured patients at all sites, local financial assistance programmes generally covered most expenses.

### Retention in PrEP care

**Figure 1:**
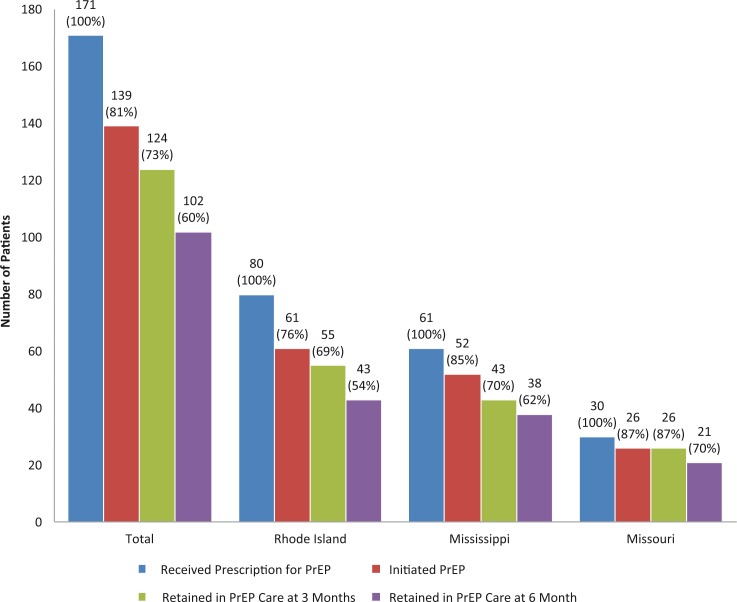
Retention in HIV pre-exposure prophylaxis (PrEP) care cascade overall and for Rhode Island, Mississippi and Missouri. Blue bars indicated the number of patients who received a prescription for PrEP (and had been in the programme for six or more months), red bars indicate the number who initiated PrEP (confirmed to have started the medication), green bars indicate the number who were retained in PrEP care at three months and purple bars indicate the number who were retained in PrEP care at six months.

Among individuals prescribed PrEP, 73% overall (69% in Rhode Island, 70% in Mississippi, 87% in Missouri) were retained in care three months after their initial prescription. Of those originally prescribed PrEP, 60% were retained in care at six months (Figure 1 and Table 2).

**Table 2:** Follow-up status for participants prescribed PrEP at least six months prior

	Providence, RI	Jackson, MS	St. Louis, MO	
	(*N*=80)	(*N*=61)	(*N*=30)	*p*-value
**Three-month follow-up status**				
**Still in care**	**54 (67.5%)**	**43 (70.5%)**	**26 (86.7%)**	**0.12**
Never started	19 (23.8%)	9 (14.8%)	4 (13.3%)	
Lost to follow-up	0	7 (11.5%)	0	
Side effects	3 (3.8%)	0	0	
Moved out of state	1 (1.3%)	2 (3.3%)	0	
Behaviour change	1 (1.3%)	0	0	
Seroconversion	1 (1.3%)	0	0	
Other	1 (1.3%)	0	0	
**Three-month adherence**				
Took four or more pills, past week	47/53 (88.7%)	34/36 (94.4%)	25/26 (96.2%)	0.49
100% adherence, past week	40/53 (75.5%)	25/36 (69.4%)	18/26 (69.2%)	0.77
100% adherence, past month	31/53 (58.5%)	16/36 (44.4%)	9/26 (34.6%)	0.12
**Six-month follow-up status**				
**Still in care**	**42 (52.5%)**	**37 (60.7%)**	**19 (63.3%)**	**0.51**
Discontinued prior to three-month visit	26 (32.5%)	18 (29.5%)	4 (13.3%)	
Lost to follow-up	7 (8.8%)	3 (4.9%)	2 (6.7%)	
Side effects	1 (1.3%)	0	0	
Moved out of state	2 (2.5%)	2 (3.3%)	1 (3.3%)	
Behaviour change	0	1 (1.6%)	2 (6.7%)	
Seroconversion	0	0	1 (3.3%)	
Other	2 (2.5%)	0	1 (3.3%)	
**Six-month adherence**				
Took four or more pills, past week	30/30 (100%)	25/31 (80.6%)	18/18 (100%)	<0.001
100% adherence, past week	27/30 (90%)	21/31 (67.7%)	16/18 (88.9%)	0.09
100% adherence, past month	23/30 (76.7%)	15/31 (48.4%)	6/18 (33.3%)	0.008

RI: Rhode Island; MS: Mississippi; MO: Missouri; PrEP: pre-exposure prophylaxis.

Among 171 patients who were given a prescription for PrEP and who were enrolled for at least six months, 139 (81%) were known to have initiated PrEP. In a multivariable model, no factors were significantly associated with initiating PrEP. Reasons for discontinuing PrEP prior to the three month visit included side effects, moving and starting PrEP but being lost to follow-up. At three months, one patient reported discontinuing PrEP because his behaviours changed and it was no longer indicated, and three additional patients reported this at six months. Among those who were still taking PrEP at the three-month visit, 18% discontinued PrEP by their six-month visit. Reasons for discontinuing by the six-month visit were similar to reasons for discontinuation prior to three months (Table 2).

In a multivariable model restricted to MSM who had started PrEP, African-American MSM had reduced odds of being retained in PrEP care at three months (aOR 0.13, 95% CI 0.02 to 0.77, *p*=0.03). No factors were associated with retention in care at six months (Table 3).

**Table 3:** Factors associated with retention in PrEP care at three and six months among MSM participants

	Started PrEP		Three-month retention in care		Six-month retention in care	
			
	Bivariate OR (95% CI)	Multivariable aOR (95% CI)	Bivariate OR (95% CI)	Multivariable aOR (95% CI)	Bivariate OR (95% CI)	Multivariable aOR (95% CI)
Age (per year)	0.99 (0.96 to 1.03)	0.97 (0.93 to 1.02)	1.05 (0.99 to 1.12)	1.03 (0.93 to 1.14)	1.02 (0.98 to 1.05)	1.00 (0.95 to 1.05)
African American/black (*vs* any other)	1.24 (0.54 to 2.82)	1.32 (0.42 to 4.15)	**0.24** **(0.08 to 0.74)**	**0.13** **(0.02 to 0.77)**	0.66 (0.30 to 1.42)	0.74 (0.25 to 2.16)
University education or above (*vs* below)	1.57 (0.72 to 3.41)	1.72 (0.71 to 4.17)	0.88 (0.28 to 2.73)	0.76 (0.19 to 3.09)	1.78 (0.82 to 3.83)	1.53 (0.63 to 3.71)
MSM (*vs* other sexual identity)	1.18 (0.36 to 3.83)	NA	2.33 (0.58 to 9.45)	NA	2.00 (0.66 to 6.07)	NA
Income (per $1000 USD)	1.01 (1.00 to 1.02)	1.01 (1.00 to 1.02)	1.00 (0.99 to 1.02)	0.99 (0.98 to 1.01)	1.01 (1.00 to 1.02)	1.01 (0.99 to 1.02)
No insurance (*vs* any)	1.36 (0.57 to 3.25)	1.42 (0.44 to 4.51)	2.64 (0.86 to 8.11)	1.48 (0.33 to 6.55)	1.17 (0.48 to 2.84)	0.87 (0.27 to 2.75)

OR, odds ratio; aOR: adjusted odds ratio; CI: confidence interval; PrEP: pre-exposure prophylaxis; MSM: men who have sex with men; USD: US dollars. Bold values indicate statistically significant results (p<0.05).

Among individuals who returned for their three-month PrEP visit, adherence (defined as taking at least four pills in the previous week) was 92%, and 72% reported perfect adherence in the previous week. Less than half of patients reported perfect adherence in the previous 30 days. Three-month adherence did not significantly vary by site. Among individuals who returned for their six-month PrEP visit, adherence was 92%, with 81% reporting perfect adherence in the past week. Fifty-six percent reported perfect adherence in the past 30 days at their six-month visit (Table 2). Reported adherence was generally similar at the three month and six month time points.

Notably, there were three seroconversions over the course of the study. One Mississippi patient tested positive for HIV before payers approved PrEP. One Rhode Island patient tested positive for HIV at his three-month visit. This patient may have been acutely infected at the time PrEP was prescribed or may have seroconverted after commencing PrEP. One Missouri patient tested positive for HIV at the six-month visit. This patient was known to be non-adherent to PrEP.

Identifying individuals at the highest risk for acquiring HIV and financial constraints are two commonly perceived barriers to implementing PrEP [19]. We were able to overcome these barriers and prescribe PrEP to diverse MSM at high risk for acquiring HIV across three clinical settings. Because a much larger fraction of patients in Mississippi was uninsured, assistance programmes were critical to facilitate PrEP uptake in this setting. Differences in insurance coverage were attributed to overall lower rates of insurance in Mississippi and Missouri and both states’ decisions not to expand Medicaid [25], which contrasts with Rhode Island's generous and early Medicaid expansion [26]. Nevertheless, it is noteworthy that patients were generally able to access medications; these findings dispel the common misperception that medication cost is a major barrier to expanding PrEP programmes. Previous reports had suggested that medication cost could be a significant barrier to successful PrEP implementation [19]. Our experience suggests that copayments or deductibles for medical services are a much greater barrier for accessing PrEP than the cost and copayments associated with the medication itself. For those without insurance, the medication can be obtained from the manufacturer's assistance programme. For individuals with insurance, medication copayments are generally covered by the manufacturer's copayment assistance programme.

These clinical programmes were moderately successfully in retaining individuals in PrEP care; nearly three-quarters were retained at three-month intervals, but this figure dropped to less than two-thirds at six months. Although not statistically significant, retention in care varied by clinical site, perhaps reflecting different demographic groups, different case management services and payer policies. Retention in care rates did not vary by a number of individual-level factors, including race, age and socio-economics including insurance coverage and income. However, African Americans were less likely to be retained in care at the three-month end point. These changes were not significant at the six-month end point, although there was also reduced power at six months. Analyses from CDC PrEP demonstration projects recently found that retention in care rates were lower among MSM of colour and these patients were less likely to have detectable blood drug levels [13]. However, our results must be interpreted with caution, as the distribution of African-American patients differed substantially by enrolment site. It is possible that other site-specific factors drove these findings. Nearly 20% of our participants were completely lost to follow-up. Given recent studies citing PrEP's efficacy in reducing HIV acquisition in real-world settings [11], our findings suggest that much greater efforts are needed to understand and enhance retention in PrEP care, particularly for MSM of colour.

Among patients retained in care, the overwhelming majority reported adherence rates commensurate with the minimum required for PrEP efficacy [24]. Our results, robust across three sites and three different socio-economic environments, demonstrated that retention in PrEP-related care may be the biggest challenge in ensuring that PrEP's protective benefits are maximized in real-world clinical settings. Although adherence has typically been considered the major limiting factor in PrEP efficacy [6,24,27], our results suggest that interventions focused on retention in care may have an equal or greater impact on PrEP's effectiveness. Interventions to improve retention will likely need to be tailored to specific economic, social and clinical environments [28,29].

Our programmes offer other important lessons. First, PrEP was successfully obtained for many high-risk individuals of low socio-economic status in three different clinical and social environments. Poverty is a well-established social determinant of the HIV epidemic in the United States [30,31]; expanding services is a priority for United States HIV prevention efforts. We were able to overcome most financial barriers to providing PrEP to participants. Although most costs were covered by insurance or patient assistance programmes, it required significant staff time to apply for these programmes and/or apply for prior authorizations. Financially sustainable PrEP programmes may depend on payer-mix and clinic-level commitments to filling gaps in insurance coverage in order to provide PrEP to economically disadvantaged persons in states with limited public insurance options. Sustaining PrEP programmes may be easier in states that expanded Medicaid under the Affordable Care Act [32], but having clinical leadership that endorses staff support of PrEP programmes is also crucial.

These results must be interpreted in the context of several limitations. Adherence measures were based on self-report and were only obtained for individuals who presented for clinical care. These individuals may have substantially different adherence patterns, and self-reported adherence may be subject to recall or reporting bias. Regression analyses focused exclusively on MSM. Lastly, we did not measure risk compensation in our sample.

## Conclusions

In three geographically diverse PrEP implementation programmes, we found suboptimal retention in care at six months across all sites due to a combination of structural and individual-level factors. Diverse strategies to pay for medications, laboratory costs and provider time, as well as interventions to promote retention in care are likely needed to reach and retain patients at highest risk for contracting HIV. PrEP support services may be even more critical for MSM of colour. Further research is needed to explore whether and how individual, social, structural or individual clinical factors may undermine or enhance uptake and subsequent retention in PrEP care.
